# A quantitative homogeneous assay for fragile X mental retardation 1 protein

**DOI:** 10.1186/1866-1955-5-8

**Published:** 2013-04-02

**Authors:** Gabi Schutzius, Dorothee Bleckmann, Sandra Kapps-Fouthier, Francesco di Giorgio, Bernd Gerhartz, Andreas Weiss

**Affiliations:** 1 Neuroscience Discovery, Novartis Pharma AG, Novartis Institutes for Biomedical Research, Postfach, Basel, CH-4002, Switzerland; 2 Developmental and Molecular Pathways, Novartis Pharma AG, Novartis Institutes for Biomedical Research, Postfach, Basel, CH-4002, Switzerland; 3 Center for Proteomic Chemistry, Novartis Pharma AG, Novartis Institutes for Biomedical Research, Postfach, Basel, CH-4002, Switzerland; 4 Present address: IRBM Promidis, Via Pontina Km 30.600, Pomezia, Roma, 00040, Italy

**Keywords:** Fragile X syndrome, FMRP, Time-resolved Förster’s resonance energy transfer, Immunoassay

## Abstract

**Background:**

Hypermethylation of the fragile X mental retardation 1 gene *FMR1* results in decreased expression of *FMR1* protein FMRP, which is the underlying cause of Fragile X syndrome – an incurable neurological disorder characterized by mental retardation, anxiety, epileptic episodes and autism. Disease-modifying therapies for Fragile X syndrome are thus aimed at treatments that increase the FMRP expression levels in the brain. We describe the development and characterization of two assays for simple and quantitative detection of FMRP protein.

**Method:**

Antibodies coupled to fluorophores that can be employed for time-resolved Förster’s resonance energy transfer were used for the development of homogeneous, one-step immunodetection. Purified recombinant human FMRP and patient cells were used as control samples for assay development.

**Results:**

The assays require small sample amounts, display high stability and reproducibility and can be used to quantify endogenous FMRP in human fibroblasts and peripheral blood mononuclear cells. Application of the assays to FXS patient cells showed that the methods can be used both for the characterization of clinical FXS patient samples as well as primary readouts in drug-discovery screens aimed at increasing endogenous FMRP levels in human cells.

**Conclusion:**

This study provides novel quantitative detection methods for FMRP in FXS patient cells. Importantly, due to the simplicity of the assay protocol, the method is suited to be used in screening applications to identify compounds or genetic interventions that result in increased FMRP levels in human cells.

## Background

With an estimated prevalence of 1 in 4,000, Fragile X syndrome (FXS) is the most prevalent monocausal inherited mental retardation disorder [1]. The disorder’s clinical symptoms include intellectual and cognitive impairment, hyperactivity and increased overall anxiety as well as early-life epilepsy episodes and autism [2]. The underlying causative mutation in almost all FXS patients is the expansion of a CGG triplet repeat expansion in the 5′ UTR of the fragile X mental retardation 1 gene *FMR1*, correlating with hypermethylation of the repeat region and the upstream *FMR1* promoter. This epigenetic modification in turn results in transcriptional silencing with reduced expression levels of *FMR1* mRNA and its protein product, the *FMR1* protein FMRP [3]. In addition to the triplet repeat expansion in the noncoding region, a small subset of about 2% of all FXS cases are caused by mutations in the coding region of the gene that also result in reduced or absent FMRP protein expression [4,5].

FMRP is a 71 kDa predominantly cytoplasmatic RNA binding protein [6] with high expression levels in the postsynaptic regions of the central nervous system, where it is associated with polyribosomes and represses the local translation of mRNAs [7]. Absence or decreased postsynaptic FMRP levels in turn lead to hyperactivity of metabotropic glutamate receptor 5 (mGluR5)-mediated synaptic pathways. This results in altered dendritic spines at the histological levels and the clinically observed cognitive and behavioral abnormalities in FXS patients [8]. Animal experiments with *FMR1* knockout mice with a 50% reduction in mGluR5 expression showed a rescue of behavioral deficits [9] and early findings in clinical trials suggest that behavioral deficits can be reduced by treatment with selective mGluR5 inhibitors in a subset of patients with full methylation of the *FMR1* promoter region [10]. However, despite those encouraging results, mGluR5-directed approaches remain symptomatic treatments that do not target the underlying disease cause – decreased postsynaptic levels of FMRP in the central nervous system. The further identification of disease-modifying therapies that can increase the postsynaptic FMRP levels will thus rely on the development of quantitative and screening compatible readouts.

Current reported methods for FMRP level analysis include immunohistochemical, protein immunoblot and ELISA assays [11,12]. While those techniques greatly improved our insights into FXS, they are either semiquantitative in nature or prove to be too labor intensive, containing multiple protocol steps that limit their use for screening of larger compound and genomic libraries in a microtiter plate format. To circumvent these limitations, we developed an alternative high-throughput compatible robust and sensitive endogenous FMRP quantification immunoassay utilizing time-resolved Förster’s resonance energy transfer (TR-FRET). TR-FRET technology is based on simultaneous in-solution binding of two fluorophore-labeled antibodies to their antigen. Antibody binding to their antigen brings the fluorophores in close enough proximity for a TR-FRET to occur after excitation of the donor fluorophore. This method has the advantages that, due to its homogeneous nature, only a single pipetting step is needed for detection and the use of low sample volumes greatly facilitates microtiter screens aimed at identifying compounds or genes that modify target protein levels. Indeed, similar methods based on TR-FRET immunoassay detection have been previously reported in protein-level modifying screens for other neurological disorders such as Parkinson’s disease [13].

## Methods

### Antibodies

N-terminal-specific anti-FMRP 1C3 (catalogue number Mab2160) was obtained from Millipore (Billerica, MA, USA), and N-terminal-specific anti-FMRP (M03) clone 3E11 (catalogue number H00002332-M03) was obtained from Abnova (Taipei City,Taiwan). C-terminal-specific anti-FMRP antibody (catalogue number F4055) was purchased from Sigma-Aldrich (St. Louis, MO, USA). Antibody labeling with Lumi4®-Tb (−Tb) or d2 (−d2) fluorophores was performed by Cisbio Bioassays (Codolet, France).

### Epitope mapping

Epitope mapping for TR-FRET antibodies was performed by peptide blot. The peptide blot covering the FMRP sequence with peptides 20 amino acids long with a seven amino acid overlap was obtained from Jpt (Berlin, Germany). After reactivation according to the manual, the membrane was blocked with 5% skim milk powder in Tris-buffered saline Tween-20 (TBST) and incubated with anti-FMRP antibody from Millipore (1:500 in 0.5% skim milk in TBST) overnight. The membrane was washed three times and incubated with secondary antibody for 1 hour and developed using enhanced chemiluminescence (GE Healthcare, Fairfield, CT, USA).

### Production, purification and characterization of recombinant FMRP

*Escherichia coli* strain BL21(DE3) harboring the expression plasmid for MBP-FMRP was cultivated at 37°C in Terrific Broth (TB) medium modified with 4-Morpholinepropanesulfonic acid (MOPS), and supplemented with 100 μg/ml ampicillin. MBP-FMRP induction was started by adding 0.5 mM IPTG at an OD_600_ of 0.55 overnight at 16°C. Cells were harvested by centrifugation and all subsequent purification steps were done at room temperature using the Äkta Avant system (GE Healthcare, Fairfield, CT, USA). Cells were resuspended in 500 ml of 50 mM Tris/HCl buffer, pH 8.0, containing 150 mM NaCl and 1 mM Tris(2-carboxyethyl)phosphine hydrochloride (TCEP). Then 1 μl Benzonase (Sigma-Aldrich (St. Louis, MO, USA)) was added with complete protease inhibitor tabs (Roche Diagnostics AG, Rotkreuz, Switzerland). Cells were ruptured using a French press and the homogenate was centrifuged at 8000×*g* for 30 minutes at 4°C. The sample was loaded on 3 ml MBP-Trap HP column (GE Healthcare) and the column was washed with 50 mM Tris/HCl, pH 8.0, buffer containing 150 mM NaCl and 1 mM TCEP. MBP-FMRP protein was eluted with 50 mM Tris/HCl, pH 8.0, buffer containing 10 mM Maltose, 150 mM NaCl and 1 mM TCEP. Fractions containing the protein were pooled and concentrated using Amicon concentrators with 30 kDa cutoff size (Millipore). Finally, MBP-FMRP solution was applied to a size exclusion chromatography column (Superdex 200, HiLoad 16/60; GE Healthcare), equilibrated with 50 mM Tris/HCl, pH 8.0, buffer containing 1 M NaCl, 1 mM TCEP, 1 mM ethylenediamine tetraacetic acid and 5% glycerol at a flow rate of 1 ml/minute. The fractions containing the purified protein were pooled. The pooled solution was analyzed by SDS-Coomassie blue gel, immunoblotting and HPLC. A Poros R1 column (Agilent Technologies, Inc., Santa Clara, CA, USA) with a flow rate of 1.2 ml/minute was used for HPLC with buffer A being H_2_O with 0.1% TFA and buffer B being acetonitrile with 0.1% TFA. MBP-FMRP was eluted by increasing the acetonitrile concentration from 5 to 95% in 6.7 minutes with a pressure limit of 170 bar.

### SDS-PAGE and immunoblot analysis

Cells were lysed in M-PER® lysis buffer (Pierce Biotechnology, Inc., Rockford, IL, USA) supplemented with 150 mM NaCl and Protease Inhibitors (Roche Diagnostics AG, Rotkreuz, Switzerland). After protein quantification with BCA Assay (Pierce Biotechnology, Inc.), equal amounts of protein were loaded on NuPage 4 to 12% Bis–Tris gels and electrophoresis was performed according to the manual. The gel was blotted on a polyvinylidene fluoride membrane (Immobilon-P; Millipore), blocked with 5% skim milk powder in TBST for 1 hour at room temperature and incubated overnight with primary antibodies. Membranes were washed and incubated with secondary antibody for 1 hour and were developed using enhanced chemiluminescence (GE Healthcare).

### Cellular models

Hek293T cells were grown at 37°C in 5% CO_2_ in Dulbecco’s minimum essential medium (DMEM + GlutaMAX) supplemented with 10% fetal bovine serum (Life Technologies Ltd, Paisley, UK). For FMRP overexpression, cells were transiently transfected with Myc-DDK-tagged FMRP (OriGene, Rockville, MD, USA) using Lipofectamine 2000 (Life Technologies Ltd). Primary human fibroblasts of healthy control patients (ID: BJ1) or FXS patients (ID: GM 09497) were obtained from ATCC (Manassas, VA, USA) and Coriell (Camden, NJ, USA) respectively and cultured in Eagle’s minimum essential medium (Sigma-Aldrich) supplemented with 15% fetal bovine serum, GlutaMAX, penicillin and streptomycin (Life Technologies Ltd).

### Time-resolved Förster’s resonance energy transfer

For TR-FRET, 5 μl sample/well was loaded on a 384-well low-volume polystyrene microtiter plate (Greiner Bio-One GmbH, Kremsmünster, Austria). After addition of 1 μl antibody solution (50 mM NaHPO_4_, 400 mM NaF, 0.1% BSA, and 0.05% Tween-20) containing Millipore Anti-FMRP-Tb antibody in combination with Abnova Anti-FMRP-d2 or Sigma Anti-FMRP-d2 in the indicated concentrations per well, plates were incubated for 20 hours at room temperature. Time-resolved FRET was determined using an Envision reader (Perkin Elmer, Inc., Waltham, MA, USA). After excitation at 320 nm, antigen-specific energy transfer was calculated as the ratio of the emission of the acceptor fluorophore d2 (665 nm) and that of the donor fluorophore Tb (620 nm). FMRP levels are presented as ΔF values, which normalize the emission of the TR-FRET signal of the acceptor fluorophore (665 nm) to that of the FRET-independent donor fluorophore (620 nm) taking into account the background fluorescence of the assay for both channels. ΔF is therefore the percentage signal over assay buffer background and is given by the equation:

$$\mathrm{\Delta F}=100*\frac{\mathrm{Rati}o_{665/620_{\mathrm{Sample}}}-\mathrm{Rati}o_{665/620_{\mathrm{Backgroundblank}}}}{\mathrm{Rati}o_{665/620_{\mathrm{Backgroundblank}}}}$$

### Time-resolved Förster’s resonance energy transfer assay in 384-well plate format

For direct high-throughput compatible quantification of endogenous FMRP levels in human fibroblasts, cells were plated the previous day at indicated cell numbers into 384-well plates (Greiner Bio-One GmbH). Medium was removed and directly lysed without prior washing with 6.5 μl M-PER lysis buffer (Pierce Biotechnology, Inc.) supplemented with 150 mM NaCl and Protease Inhibitors (Roche Diagnostics AG). For detection of endogenous FMRP levels in human peripheral blood mononuclear cells (PBMCs), PBMC pellets were lysed with M-PER lysis buffer supplemented with 150 mM NaCl and protease inhibitors for 30 minutes at 4°C under shaking. For both cell types, 5 μl lysate was then transferred into white, opaque 384-well low-volume polystyrene microtiter plates (Greiner Bio-One GmbH) and TR-FRET quantification was performed as described above.

### Limit of detection determination

Limit of detection (LoD) levels for the indicated TR-FRET antibody assays were determined according to guidelines from the Clinical and Laboratory Standards Institute [14]. The LoD is defined as:

$$\mathrm{LoD}=\mathrm{LoB}+1.645*{\mathrm{SD}}_{\mathrm{lowest}\;\mathrm{concentration}\;\mathrm{sample}}$$

This LoD describes the lowest analyte concentration, reliably being distinguishable from the limit of blank (LoB):

$$\mathrm{LoB}={\mathrm{mean}}_{\mathrm{blank}}+1.645*{\mathrm{SD}}_{\mathrm{blank}}$$

The LoB was determined by measurement of a minimum of 20 replicates containing assay buffer and antibodies. The LoD was then quantified with repeated measurements of the indicated concentrations of purified FMRP for which ≥95% of the observed signal was higher than the LoB corresponding to the used detection antibody pair.

### Statistical analysis

FMRP levels are presented as averages with standard deviations. The Z´-factor was calculated according to [15].

## Results and discussion

To aid in the development of a homogeneous TR-FRET immunoassay for FMRP we first generated the isolated protein by bacterial expression and subsequent column purification. Purified FMRP has been previously reported to be prone for aggregation and precipitation, limiting its use as a stable protein standard. These protein instabilities can be addressed by fusing the maltose binding protein MBP to the FMRP N-terminus [11]. We thus opted to use MBP-FMRP protein as our isolated analyte standard. Purity and concentration or MBP-FMRP was determined with SDS-gel, protein immunoblot and HPLC (Additional file 1: Figure S1). The purified protein was used in the following assay development steps.

**Figure 1:**
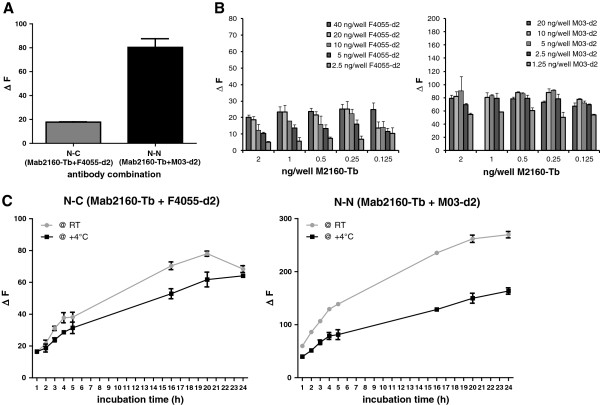
**Assay condition optimization for detection of human FMRP by time-resolved Förster’s resonance energy transfer. (A**) Purified recombinant FMRP (5 ng) in a 1 ng/μl concentration were analyzed by two different time-resolved Förster’s resonance energy transfer (TR-FRET) immunoassays using an antibody combination detecting a N-terminal and a C-terminal FMRP epitope (N-C, Mab2160-Tb + SigmaF4055-d2) or a combination of antibodies in which both detect N-terminal epitopes (N-N, Mab2160-Tb + M03-d2). N-N antibody combination resulted in stronger signal over background detection. **(B**) Antibody titer optimization for N-C and N-N combination for detection of purified recombinant FMRP (5 ng). **(C**) Optimization of TR-FRET assay conditions for time and temperature of incubation. Both assays performed best at room temperature incubation overnight (≥20 hours). All values presented as percentage signal over assay buffer background. All data and error bars represent averages and standard deviations of triplicates.

A previous publication reports the successful use of two defined FMRP epitopes for detection using an ELISA method [11]. We thus next tested whether antibodies against these described epitopes (Mab2160 raised against a N-terminal epitope, F4055 raised against a C-terminal epitope) would also be functional when labeled and utilized in a TR-FRET immunoassay setup. Using this antibody combination in an unoptimized protocol with 1 ng/well Mab2160-Tb and 10 ng/well F4055-d2 incubation for 2 hours at room temperature, a small but FMRP-specific detection signal was observed when using 1 ng/μl purified MBP-FMRP as antigen (Figure 1A, N-C). Since the signal strength of TR-FRET detection is closely related to the distance of the antigen-binding antibodies, we asked ourselves whether substitution of the C-terminal F4055-d2 acceptor antibody against a more N-terminally binding antibody would result in a stronger TR-FRET signal in combination with the N-terminal Mab2160 antibody. Since the epitope for Mab2160 has not yet been defined in detail and to avoid testing a N-terminal antibody combination that competed for the same epitope, we thus performed epitope mapping for Mab2160 using a peptide blot spanning the whole human FMRP amino acid sequence (Additional file 2: Figure S2).

After having identified the Mab2160 binding region on FMRP as amino acids 34 to 39, we proceeded to combine the antibody with the N-terminally binding antibody Abnova-M03 whose described epitope (amino acids 121 to 220) is in proximity to but is not overlapping with Mab2160. This antibody combination indeed resulted in a stronger and specific FMRP detection signal (Figure 1A, N-N). We continued to perform all further assay optimization and characterization steps with both antibody combinations in order to establish two different TR-FRET detection assays for FMRP.

The antigen-specific TR-FRET signal is determined as the ratio of the emission wavelengths specific for the acceptor fluorophore d2 (665 nm) and the donor fluorophore Tb (620 nm) coupled to the respective antibodies used. Using purified MBP-FMRP as analyte, we thus first optimized the assay conditions for the titers of donor-acceptor antibodies per well (Figure 1B). Having identified the ideal antibody concentrations for the N-C-terminal (0.5 ng/well Mab2160-Tb + 20 ng/well F4055-d2) as well as the N-N-terminal antibody combination (0.5 ng/well Mab2160-Tb + 5 ng/well M03-d2), we proceeded to assess the assay kinetics for incubation temperature and time (Figure 1C).

**Figure 2:**
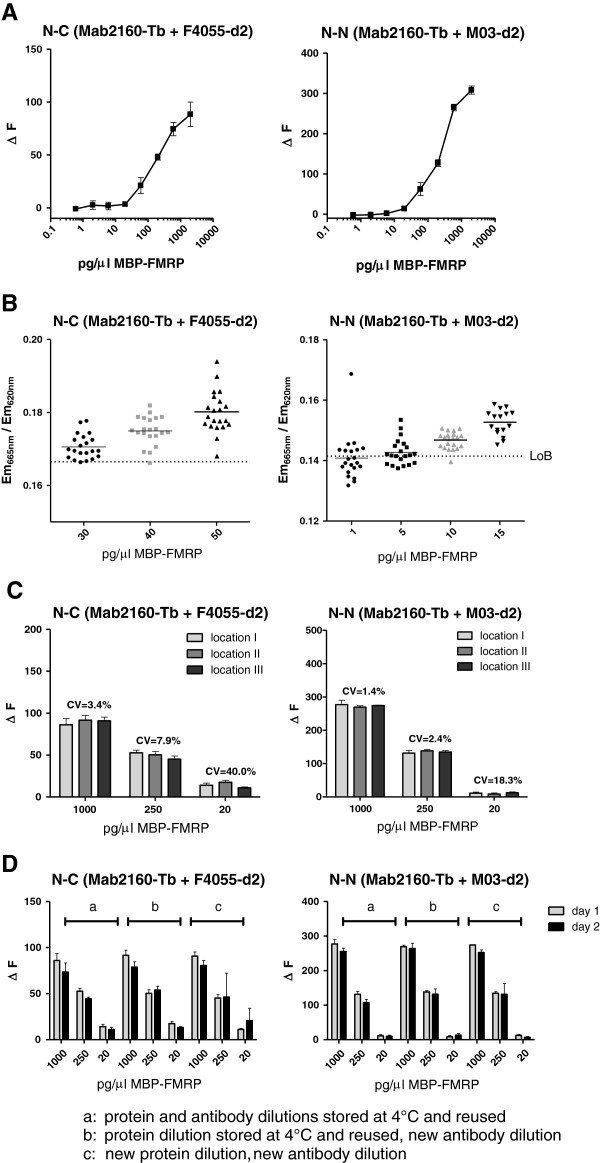
**TR-FRET assay characterization for reliability, robustness and reproducibility. (A)** Dynamic range of both time-resolved Förster’s resonance energy transfer (TR-FRET) assays was assessed by serial dilutions of purified recombinant FMRP starting at 2,000 pg/μl into assay buffer in wells of low-volume 384-well plates. **(B)** Limits of detection for both assays were determined according to the standards set by the Clinical and Laboratory Standards Institute [12]. The TR-FRET signal intensity for FMRP protein concentrations around the expected putative limits of detection (as previously examined in the dynamic range assessment) were compared with the limit of blank for each assay. Limit of detection was defined as the FMRP concentration at which ≥95% of analyzed samples resulted in a TR-FRET signal above the limit of blank. Determined limit of detection concentrations for each assay are indicated by grey symbols. **(C)** Assessment of intra-assay variability for each assay with three different FMRP concentrations distributed randomly across a low-volume 384-well plate (locations I to III). **(D)** Assessment of inter-assay variability was determined for each assay by testing reused frozen and thawed protein standard and antibodies (a), reused protein standard but fresh antibody detection solution (b) or freshly diluted protein standard and antibody solution (c) on two independent days. All values for **A**, **C** and **D** are presented as percentage signal over assay buffer background. All data and error bars represent averages and standard deviations of triplicates.

After optimization of these parameters, the assay performance was characterized for dynamic range, lower limit of detection as well as intra-assay and inter-assay stability. Dilution series with purified protein showed a dynamic range of about three orders of magnitude for both assays with linear signals up to 2,000 pg/μl MBP-FMRP (Figure 2A). The LoD for the assays was determined by assessing the protein concentration by which 95% of samples delivered a signal above the assay buffer LoB [14]. The LoD was calculated as 40 pg/μl for the N-C-terminal antibody combination and 10 pg/μl MBP-FMRP for the N-N-terminal antibody combination (Figure 2B). High intra-plate and inter-plate stability of the assays – a prerequisite for high-throughput screening – was verified by distributing technical replicates of different MBP-FMRP dilutions across random wells of 384-microtiter plates and repeating the assay on independent days with either stock or fresh dilutions of antigen and antibodies (Figure 2C,D).

After these assay characterization steps with purified antigen, we proceeded to optimize the lysis buffer conditions for FMRP protein expressed in cells. We transfected HEK293 cells with human FMRP or mock plasmids and verified FMRP overexpression over endogenous FMRP levels present in HEK cells by protein immunoblot (Additional file 3: Figure S3A). Equal amounts of mock and FMRP transfected HEK cells were then lysed with six different lysis buffers and the FMRP signal intensity was determined using the N-N-terminal antibody TR-FRET pair (Additional file 3: Figure 3B).

**Figure 3:**
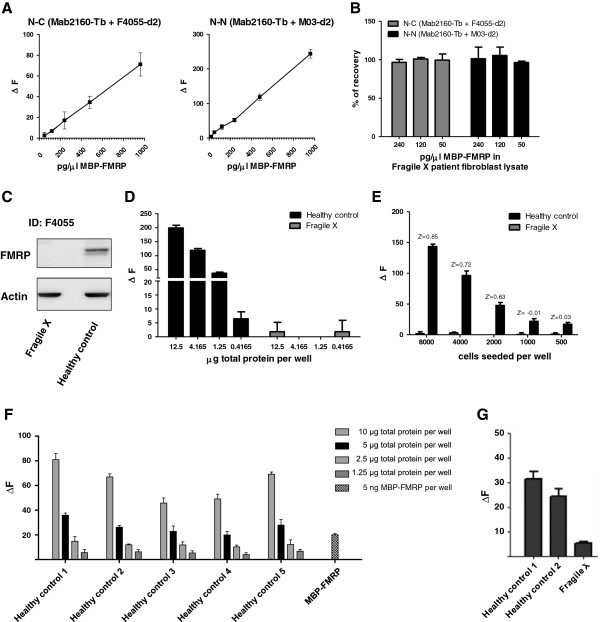
**Assay performance and application for endogenous human FMRP in fibroblasts. (A)** Determination of time-resolved Förster’s resonance energy transfer (TR-FRET) assay linearities for purified FMRP when spiked into a fibroblast lysate from a Fragile X syndrome (FragileX) patient without any detectable endogenous FMRP levels. **(B)** Recovery rate calculations of FMRP protein in FragileX patient lysate based on expected versus quantified FMRP values. **(C)** Immunoblot for endogenous human FMRP protein in lysates from a healthy control or a FragileX patient. **(D)** Detection of endogenous human FMRP by the N-N-antibody TR-FRET assay in 5 μl sample volume in low-volume 384-well plate format for serial dilutions of lysates from a healthy control and a FragileX patient. **(E)** Quantification of endogenous FMRP in healthy control versus FragileX patient fibroblast lines grown and lysed in 384-well plates. **(F)** Detection of endogenous FMRP in peripheral blood mononuclear cell (PBMC) lysates of healthy human control volunteers by the N-N-antibody combination in the low-volume 384-well plate format. Total protein concentrations per well are indicated. Purified MBP-FMRP protein (5 ng) was used as control. **(G)** Comparison of FMRP detection signal in PBMC lysates of healthy control patients versus PBMC lysate obtained from a FragileX patient (1.65 μg total protein loaded per well for each sample). Values for A, D, E, F and G presented as percentage signal over assay buffer background. All data and error bars represent averages and standard deviations of triplicates.

After optimization of the lysis conditions for efficient FMRP extraction from cells and TR-FRET assay compatibility, we determined the linearities and recovery rates for FMRP in a relevant biological matrix. We thus spiked recombinant FMRP protein into lysate of FXS patient-derived fibroblasts. Both assays exhibited excellent linearity and recover rates for human FMRP protein (Figure 3A,B) when spiked into a complex biological matrix void of detectable endogenous FMRP protein levels (Figure 3C). Next, we investigated whether the more sensitive N-N-terminal antibody combination was capable of robustly quantifying endogenous human FMRP levels from batch lysates of a healthy control over a FXS patient-derived fibroblast line (Figure 3D). Endogenous FMRP was readily detected in healthy human control fibroblasts line when compared with FXS patient cells. Having determined the proof-of-principle that the optimized homogeneous TR-FRET assay can be used to accurately detect endogenous FMRP protein in batch lysates of human material, we assessed whether detection was also feasible in fibroblasts grown and lysed directly in a 384-well microtiter plate, a format commonly used for compound or genetic screens (Figure 3E). Z′-factor values of 0.85 for 8,000 seeded cells/well and 0.72 for 4,000 seeded cells/well indicated the assay’s capability to be used in unbiased screening efforts to identify treatments aimed at increasing FMRP levels in FXS patient-derived fibroblasts.

Finally, to determine whether the method is also suitable for the detection of endogenous FMRP in PBMCs, a cellular population easily accessible in clinical practice, we next analyzed serial dilutions of human PBMC lysates from healthy control volunteers. In agreement with the FMRP detection in human fibroblasts, endogenous FMRP was readily quantified in all PBMC lysates from healthy control subjects in a linear manner (Figure 3F) whereas FMRP detection signal was markedly reduced in a PBMC preparation of a FXS patient (Figure 3G).

Some caveats of the assay require further investigation in future studies. First, FXS is a neurological retardation disorder with the underlying pathomechanism likely to be a result of decreased postsynaptic levels of FMRP [7], which in turn are caused by hypermethylation of the *FMR1* promoter [3]. It is currently unknown whether this epigenetic modulation pattern or the enzymatic machinery required for possible demethylation is comparable in post-mitotic neuronal cell populations and proliferating cells such as the fibroblasts used in this study. Future studies using FXS patient fibroblast iPS-derived neurons could help to better understand this question and to elucidate whether screening for FMRP level increase in fibroblast lines will translate into efficacy in neurons. Second, while the signal-to-background value and Z′ factor of our assay can be assessed by comparing endogenous wild-type FMRP expression levels in healthy control fibroblasts versus FXS patient-derived fibroblasts, a more informative comparison would have been to assess the effect of an established tool compound known to increase FMRP levels in FXS patient cells versus dimethyl sulfoxide mock control. Indeed, SIRT1 inhibition by splitomicin and treatment with the cytosine nucleoside analogue 5-azacytidine have been reported to increase *FMR1*/FMRP levels in patient cells [16,17]. However, a closer analysis of the reported data limited their use as tool compounds for a screening format for the following reasons. First, the significant increases of FMRP levels with those treatments have only been achieved by using prolonged treatment durations of 3 to 10 days with constant renewal of cell culture medium during culture. Such a repeated medium change with diluted compounds and long culture times of a proliferative cell line is not suitable in the 384-well microtiter plate high-throughput screening mode. Second, even when treating for these prolonged periods, the reported increase in *FMR1* transcript levels in FXS patient cell lines reached only up to ~20% of healthy control levels. Indeed, 24-hour 5-azacytidine treatment which is more comparable and amendable to screening conditions only resulted in an increase of *FMR1* levels to 4% of that of healthy controls, a change likely to small to be detected in a microtiter plate format with statistical significance [16]. Third, the observed maximum treatment effect of up to 20% of normal levels was restricted to some lymphoblast lines from selected patients while the effect was much lower or even absent in other lymphoblast or fibroblast cell lines derived from other FXS patients, a result attributed to differential methylation status of the patients *FMR1* promoter regions. It should be pointed out that these above-described limitations can be attributed to so far unanswered biological questions in the FXS field and are not limitations resulting from the intrinsic technical principle of our assay. We thus feel confident that with the continuously increasing knowledge for this devastating neurological disorder, these open questions will be addressed.

## Conclusion

In summary, we describe the development of novel microtiter TR-FRET immunoassays for the quantification of FMRP. These assays are robust, reliable and reproducible and require only small sample volumes for quantification of endogenous FMRP in human cells. Importantly, the assays distinguish themselves from alternative FMRP immunoassays that require sequential and labor-intensive capture and detection steps, which renders them impractical for high-throughput screens. Owing to the simplicity of the TR-FRET assay protocol, the herein described assays are suitable for medium-throughput to high-throughput screening efforts that aim to find genetic modifiers or compounds that increase FMRP levels in FXS patient-derived cell lines.

## Abbreviations

ELISA: Enzyme-linked immunosorbent assay; FMR1: Fragile X mental retardation 1; FMRP: *FMR1* protein; FXS: Fragile X syndrome; HPLC: High-performance liquid chromatography; LoB: Limit of blank; LoD: Limit of detection; mGluR5: Metabotropic glutamate receptor 5; PBMC: Peripheral blood mononuclear cell; TBST: Tris-buffered saline Tween-20; TCEP: Tris(2-carboxyethyl)phosphine hydrochloride; TR-FRET: Time-resolved Förster’s resonance energy transfer; UTR: Untranslated region

## Competing interests

GS, DB, SK-F, FdG and BG are employees of Novartis Pharma AG, Switzerland. AW is an employee of IRBM Promidis, Italy.

## Authors’ contributions

GS carried out the epitope mapping, TR-FRET assay development and performed the data analysis. DB and FdG provided the FXS patient fibroblast cultures. SK-F and BG carried out the bacterial culture and performed the purification and characterization of the recombinant FMRP. AW and GS designed the experiments and drafted the manuscript. All authors read and approved the final manuscript.

## Supplementary Material

Additional file 1: Figure S1 Purification and characterization of recombinant MBP-FMRP protein. (A) Bacterial expressed MBP-FMRP protein was analyzed by Coomassie blue staining on SDS-PAGE gel. Two main bands are visible after pooling fractions eluted from a MBP-Trap HP column. (B) MBP-FMRP protein analyzed by immunoblot with C-terminal anti-FMRP (Sigma-Aldrich, St. Louis, MO , USA) and N-terminal anti-FMRP (Abnova , Taipei City, Taiwan). Both main bands identified via Coomassie gel represent purified recombinant FMRP protein, with the lower band being a proteolytic N-terminal fragment only detectable by the N-terminal antibody. (C) HPLC analysis of protein verifies high purity of the recombinant FMRP isolated from bacterial cultures. Click here for file

Additional file 2: Figure S2 Mapping of the N-terminal Mab2160 epitope. (A) A peptide blot covering the whole human FMRP sequence in peptides 20 amino acids long with a seven amino acid overlap was subjected to Mab2160 incubation with subsequent immunoblot detection. (B) Mab2160 epitope was identified as amino acids 34–39 (NNWQPD). Click here for file

Additional file 3: Figure S3 Optimization of cell lysis conditions for subsequent TR-FRET detection of human FMRP protein. (A) Human FMRP protein was transiently expressed in HEK293 cells. Increased expression over endogenous levels was verified with immunoblot. (B) Equal amounts of FMRP or mock transfected HEK293 cells were lysed with different lysis buffers as indicated. Lysates were analyzed by TR-FRET for FMRP levels after normalization to total protein content. Click here for file
