# Supplementary material for: A quantitative homogeneous assay for fragile X mental retardation 1 protein
Source: J Neurodev Disord. 2013 Apr 2;5(1):8. doi: 10.1186/1866-1955-5-8 (PMC3635944; doi:10.1186/1866-1955-5-8)
Supplement: Additional file 1: Figure S1 — Purification and characterization of recombinant MBP-FMRP protein. (A) Bacterial expressed MBP-FMRP protein was analyzed by Coomassie blue staining on SDS-PAGE gel. Two main bands are visible after pooling fractions eluted from a MBP-Trap HP column. (B) MBP-FMRP protein analyzed by immunoblot with C-terminal anti-FMRP (Sigma-Aldrich, St. Louis, MO , USA) and N-terminal anti-FMRP (Abnova , Taipei City, Taiwan). Both main bands identified via Coomassie gel represent purified recombinant FMRP protein, with the lower band being a proteolytic N-terminal fragment only detectable by the N-terminal antibody. (C) HPLC analysis of protein verifies high purity of the recombinant FMRP isolated from bacterial cultures. [file 1866-1955-5-8-S1.pdf]

Supplementary Figures

Supplementary Figure 1

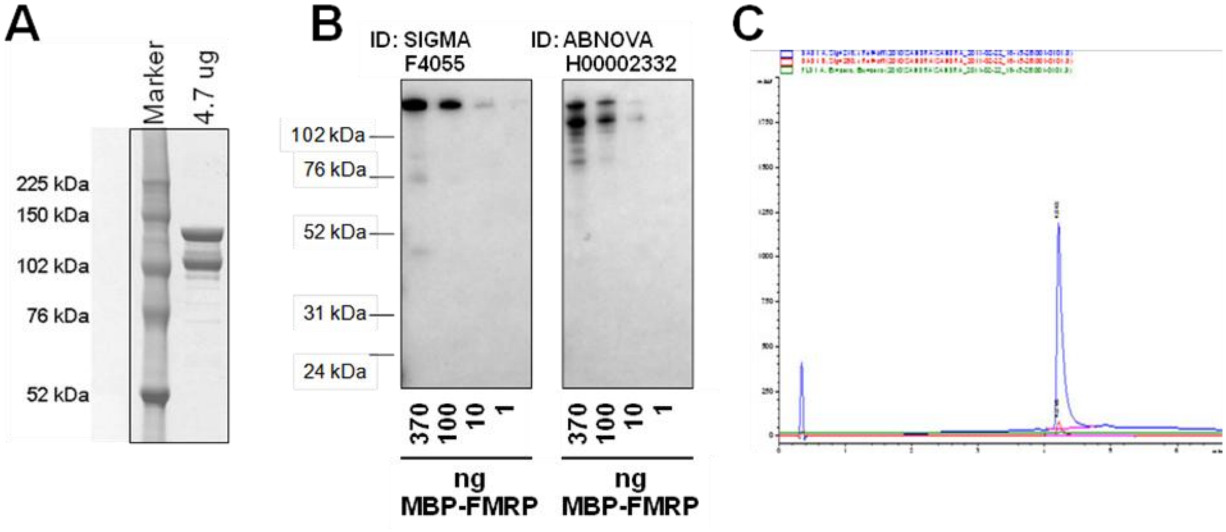

Supplementary Figure 1: Purification and characterization of recombinant MBP-FMRP protein

A) Bacterial expressed MBP-FMRP protein was analysed by coomassie blue staining on a SDS-PAGE gel. Two main bands are visible after pooling fractions eluted from a MBP-Trap HP column. B) MBP-FMRP protein analysed by immunoblot with C-terminal anti-FMRP (Sigma) and N-terminal anti-FMRP (Abnova). Both main bands identified via coomassie gel represent purified recombinant FMRP protein with the lower band being a proteolytic N-terminal fragment only detectable by the N-terminal antibody. C) HPLC analysis of protein verifies high purity of the recombinant FMRP isolated from bacterial cultures.
