# Supplementary material for: A quantitative homogeneous assay for fragile X mental retardation 1 protein
Source: J Neurodev Disord. 2013 Apr 2;5(1):8. doi: 10.1186/1866-1955-5-8 (PMC3635944; doi:10.1186/1866-1955-5-8)
Supplement: Additional file 2: Figure S2 — Mapping of the N-terminal Mab2160 epitope. (A) A peptide blot covering the whole human FMRP sequence in peptides 20 amino acids long with a seven amino acid overlap was subjected to Mab2160 incubation with subsequent immunoblot detection. (B) Mab2160 epitope was identified as amino acids 34–39 (NNWQPD). [file 1866-1955-5-8-S2.pdf]

Supplementary Figure 2

A

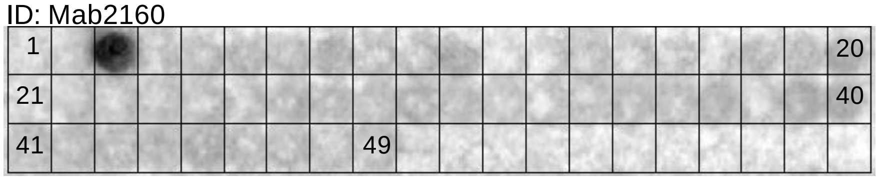

B

Peptide ID	Starting aminoacid	Peptide sequence	Peptide ID	Starting aminoacid	Peptide sequence	Peptide ID	Starting aminoacid	Peptide sequence
1	1	MEELVVEVRGSNGAFYKAFV	21	261	TFHIYGEDQDAVKKARSFLE	41	521	ERESFLRRGDGRRRGGGGRG
2	14	AFYKAFVKDVHEDSITVAFE	22	274	KARSFLEFAEDVIQVPRNLV	42	534	RGGGGRGQGGRGRGGGFKGN
3	27	SITVAFE**<u>NNWQPD</u>**RQIPFHD	23	287	QVPRNLVGKVIGKNGKLIQE	43	547	GGGFKGNDDHSRTDNRPRNP
4	40	RQIPFHDVRFPPPVGYNKDI	24	300	NGKLIQEIVDKSGVVRVRIE	44	560	DNRPRNPREAKGRTTDGSLQ
5	53	VGYNKDINESDEVEVYSRAN	25	313	VVRVRIEAENEKNVPQEEEI	45	573	TTDGSLQIRVDCNNERSVHT
6	66	EVYSRANEKEPCCWWLAKVR	26	326	VPQEEEIMPPNSLPSNNSRV	46	586	NERSVHTKTLQNTSSEGSRL
7	79	WWLAKVRMIKGEFYVIEYAA	27	339	PSNNSRVGPNAPEEKKHLDI	47	599	SSEGSRLRTGKDRNQKKEKP
8	92	YVIEYAACDATYNEIVTIER	28	352	EKKHLDIKENSTHFSQPNST	48	612	NQKKEKPDSVDGQQPLVNGV
9	105	EIVTIERLRSVNPNKPATKD	29	365	FSQPNSTKVQRVLVASSVVA	49	625	QPLVNGVPSVDGQQPLVNGV
10	118	NKPATKDTFHKIKLDVPEDL	30	378	VASSVVAGESQKPELKAWQG			
11	131	LDVPEDLRQMCAKEAAHKDF	31	391	ELKAWQGMVPFVFVGTKDSI			
12	144	EAAHKDFKKAVGAFSVTYDP	32	404	VGTKDSIANATVLLDYHLNY			
13	157	FSVTYDPENYQLVILSINEV	33	417	LDYHLNYLKEVDQLRLERLQ			
14	170	ILSINEVTSKRAHMLIDMHF	34	430	LRLERLQIDEQLRQIGASSR			
15	183	MLIDMHFRSLRTKLSLIMRN	35	443	QIGASSRPPPNRTDKEKSYV			
16	196	LSLIMRNEEASKQLESSRQL	36	456	DKEKSYVTDDGQGMGRGSRP			
17	209	LESSRQLASRFHEQFIVRED	37	469	MGRGSRPYRNRGHGRRGPGY			
18	222	QFIVREDLMGLAIGTHGANI	38	482	GRRGPGYTSGTNSEASNASE			
19	235	GTHGANIQQARKVPGVTAID	39	495	EASNASETESDHRDELSDWS			
20	248	PGVTAIDLDEDTCTFHIYGE	40	508	DELSDWSLAPTEEERESFLR			

Supplementary Figure 2: Mapping of the N-terminal Mab2160 epitope

A) A peptide blot covering the whole human FMRP sequence in 20 amino acid long peptides with a 7 amino acid overlap was subjected to Mab2160 incubation with subsequent immunoblot detection. B) Mab2160 epitope was identified as amino acids 34-39 (NNWQPD).
