# Supplementary material for: A quantitative homogeneous assay for fragile X mental retardation 1 protein
Source: J Neurodev Disord. 2013 Apr 2;5(1):8. doi: 10.1186/1866-1955-5-8 (PMC3635944; doi:10.1186/1866-1955-5-8)
Supplement: Additional file 3: Figure S3 — Optimization of cell lysis conditions for subsequent TR-FRET detection of human FMRP protein. (A) Human FMRP protein was transiently expressed in HEK293 cells. Increased expression over endogenous levels was verified with immunoblot. (B) Equal amounts of FMRP or mock transfected HEK293 cells were lysed with different lysis buffers as indicated. Lysates were analyzed by TR-FRET for FMRP levels after normalization to total protein content. [file 1866-1955-5-8-S3.pdf]

Supplementary Figure 3

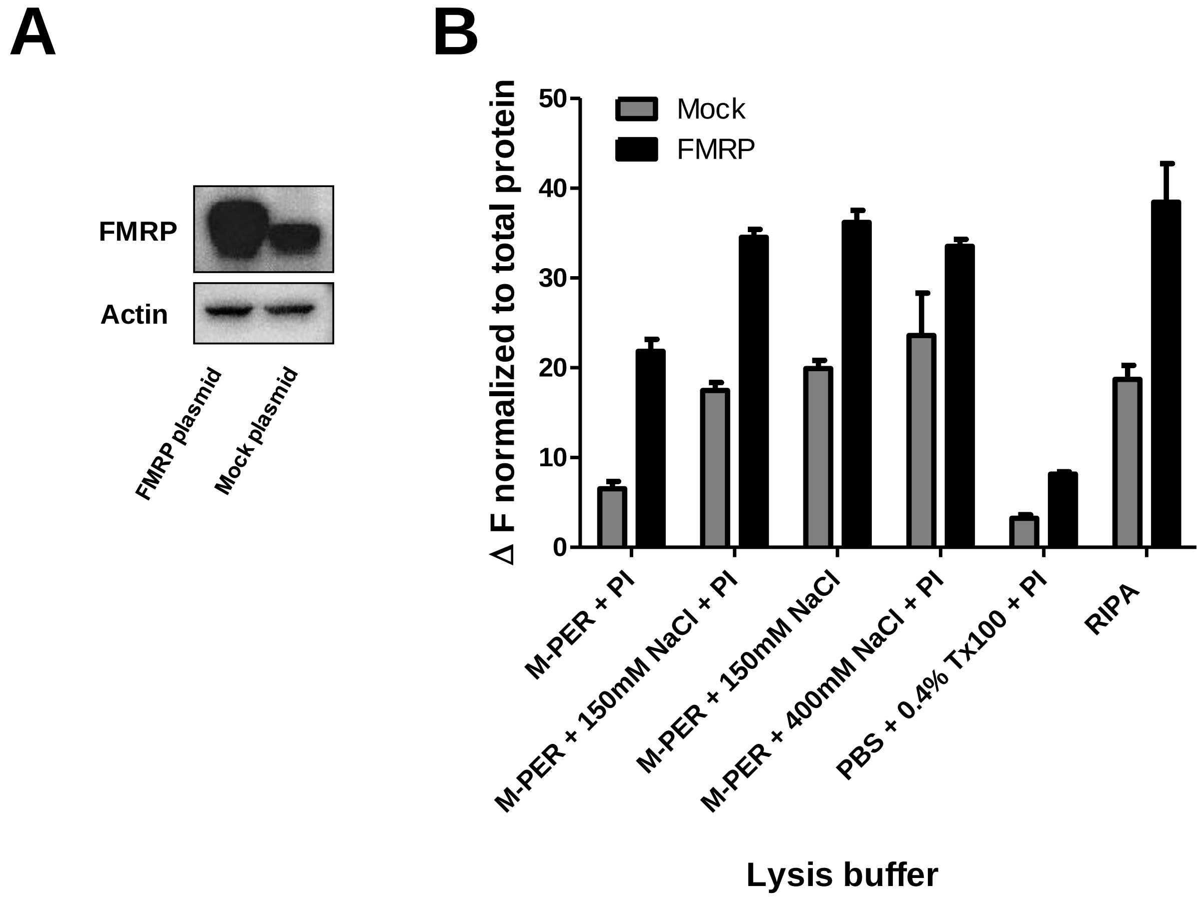

Supplementary Figure 3: Optimization of cell lysis conditions for subsequent TR-FRET detection of human FMRP protein

A) Human FMRP protein was transiently expressed in HEK293 cells. Increased expression over endogenous levels was verified with immunoblot. B) Equal amounts of FMRP or mock transfected HEK293 cells were lysed with different lysis buffers as indicated. Lysates were analysed by TR-FRET for FMRP levels after normalization to total protein content.
